# Regulatory T Cells Suppress T Cell Activation at the Pathologic Site of Human Visceral Leishmaniasis

**DOI:** 10.1371/journal.pone.0031551

**Published:** 2012-02-08

**Authors:** Ambak K. Rai, Chandreshwar P. Thakur, Amar Singh, Tulika Seth, Sandeep K. Srivastava, Pushpendra Singh, Dipendra K. Mitra

**Affiliations:** 1 Cellular Immunology Division, Department of T.I.I., All India Institute of Medical Sciences (AIIMS), Ansari Nagar, New Delhi, India; 2 Balaji Utthan Sansthan, Patna, Bihar, India; 3 Department of Hematology, All India Institute of Medical Sciences (AIIMS), Ansari Nagar, New Delhi, India; University of Palermo, Italy

## Abstract

Suppression of T cell response is thought to be involved in the pathogenesis of visceral leishmaniasis (VL). Regulatory T cell (Treg) mediated immune-suppression is reported in animal models of *Leishmania* infection. However, their precise role among human patients still requires pathologic validation. The present study is aimed at understanding the frequency dynamics and function of Treg cells in the blood and bone marrow (BM) of VL patients. The study included 42 parasitologically confirmed patients, 17 healthy contact and 9 normal bone marrow specimens (NBM). We show i) the selective accumulation of Treg cells at one of the disease inflicted site(s), the BM, ii) their *in vitro* expansion in response to *LD* antigen and iii) persistence after successful chemotherapy. Results indicate that the Treg cells isolated from BM produces IL-10 and may inhibit T cell activation in IL-10 dependent manner. Moreover, we observed significantly higher levels of IL-10 among drug unresponsive patients, suggesting their critical role in suppression of immunity among VL patients. Our results suggest that IL-10 plays an important role in suppression of host immunity in human VL and possibly determines the efficacy of chemotherapy.

## Introduction

Th1 effector response supposedly contains *Leishmania donovani* (*LD*) infection while polarized Th2 like response compromises the immune containment of *LD* infection, particularly at the pathologic site(s) of VL including bone marrow (BM) [1], [2]. Selective recruitment of phenotypically and functionally distinct T cells subsets at various proportions may determine the bulk T cell function in totality at the pathologic site(s), as in case of cutaneous leishmaniasis (CL) [3]. Effector immune response is also critical for efficacy of chemotherapeutic agent like SAG, as its conversion into active tri-valent form (Sb^III^) occurs within activated macrophages [4]–[6]. A critical unresolved issue is the state of immune suppression [6]–[8], in spite of the presence of Th1 effector immune response (IFN-γ) [9] along with IL-4 [10] and IL-10 [11] particularly at the disease site(s) among VL patients. It is therefore envisaged that simultaneous and persistent production of these suppressive cytokines constitutes the key element of well observed suppression of immune response among VL patients.

Regulatory T cells (Treg) are viewed as a major suppressor of effector T cells (Teff) and their role in local immuno-suppression in mice model of CL is documented [3]. Emergence of definitive phenotypic markers of Treg cells such as FoxP3, CD39 and CD127 enabled to study them in various infectious diseases such as human tuberculosis [12], experimental CL [3] and advanced our understanding of the role Treg cells play in containing these pathogens. Accumulation of IL-10 producing Treg cells (CD4^+^CD25^+^FoxP3^+^) at the pathologic sites of CL has been shown to induce parasitic persistence and reactivation of the pathology [3]. In contrast, some studies identified FoxP3- cells as the source of IL-10 in mice model of CL [13], [14]. Similar report on the cellular source of IL-10 among VL patients is available [15]. However, conclusive role of Treg cells in suppression of immunity in human VL is yet to be evidenced.

Here, we show a definitive enrichment of Treg cells (CD4^+^CD25^+^FoxP3^+^) among VL patients, which outnumbered that of effector T cells (CD4^+^CD25^+^FoxP3^−^) in the BM (or disease sites) of VL patients. We also demonstrate that the CD4^+^FoxP3^+^ Treg cells isolated from the disease site (BM) of VL patients are a source of IL-10 along with CD4^+^FoxP3^−^ cells. Importantly, Treg cells persisted among the patients even after successful chemotherapy. In addition, these cells proliferated well in response to *in vitro* challenge with *LD* antigens, suggesting the antigen driven expansion of Treg cells among VL patients. Interestingly, we show that SAG responsive and unresponsive patients had significantly lower and higher pre-treatment levels of IL-10 respectively, suggesting its role in immune-suppression in human VL. The present investigation highlights the important role that the Treg cells may play in the immune-pathogenesis of human VL and determine the responsiveness to conventional anti-*Leishmania* drug SAG through their suppressive influence on the local effector T cell response.

## Results

### i) Enrichment of Treg cells in the BM of VL patients

**Figure 1:**
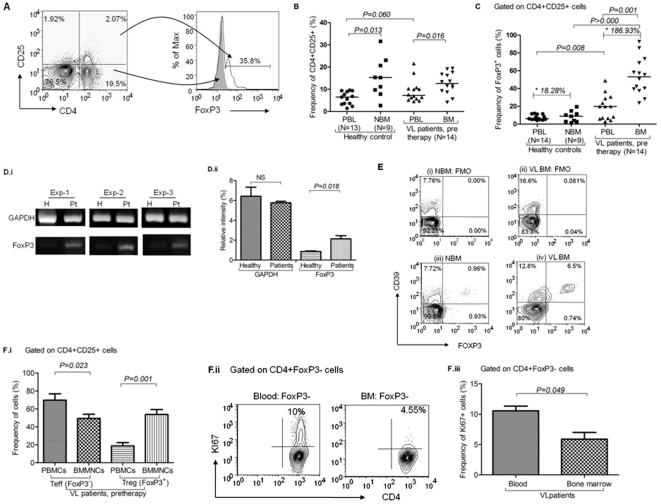
Enrichment of Treg cells at the disease site of visceral leishmaniasis (VL) patients: **A**) **Enumeration of FoxP3^+^ Treg cells:** On gated lymphocytes, expression of CD4 and CD25 was defined and subsequently expression of FoxP3 was observed among gated CD4^+^CD25^+^ cells. Overlay histogram shows FoxP3^+^ cells are exclusively present among CD4^+^CD25^+^ T cells (thin line) and absent among CD4^+^CD25^−^ population (solid line). **B**) **Increased frequency of CD4^+^CD25^+^ cells at disease sites:** Dual positive cells were enumerated among the MNCs isolated from the peripheral blood, bone marrow (BM) of VL patients (*n = 14*), peripheral blood of HCs (*n = 13*) and normal bone marrow (NBM; *n = 9*). CD4^+^CD25^+^ cells were significantly enriched in BM-MNCs as compared to PBMCs of VL patients (*p = 0.016, unpaired t test*). A statistically non-significant increase is also observed in PBMCs of VL patients in comparison with that of HCs (*p = 0.060, Unpaired t test*). Horizontal lines in dot plot depict median value. **C**) **Increased frequency of CD4^+^CD25^+^FoxP3^+^ cells in BM of VL patients:** Significant increase in frequency of CD4^+^CD25^+^FoxP3^+^ cells in PBMCs of patients (*n = 14*) as compared to that of HCs (*n = 13*; *p = 0.009*, *unpaired t test*). CD4^+^CD25^+^FoxP3^+^ cells are further enriched in BM-MNCs at disease site (*n = 15*) as compared to their autologus PBMCs (n = 14; *p = 0.001, paired t test*) and NBM (*n = 9*; *p>0.000*, *unpaired t test*). Horizontal lines in dot plot depict median values. Asterix values are enrichment ratios {ER = (Mean of Treg in BM- blood)/Mean of Treg in blood ×100) of FoxP3^+^ Treg cells in the NBM (*ER = 18.26.%*) and VL patient's BM (*ER = 186.93%*) relative to peripheral blood. **D**) **Increased expression of FoxP3 mRNA in PBMCs of patients:** (i) Photograph depicts increased expression of FoxP3 mRNA in PBMCs of VL patients than that of HCs. Three individual experiments are shown herewith. (ii) Relative density analysis shows increase in FoxP3 mRNA in patient's PBMCs (*p = 0.018, unpaired t test*). However, GAPDH expression remains unchanged in both the group. **E**) **CD4^+^FoxP3^+^CD39^+^ cells are increased in the VL-BM:** Data shows higher frequency of CD4^+^FoxP3^+^CD39^+^ cells at the disease sites of VL patients (iv; 6.5%) as compared to NBM (iii; 0.96%). Contour plots i and ii show fluorescence minus one (FMO) staining for iii and iv respectively. **F**) **FoxP3^+^ (Treg) and FoxP3^−^ (Teff) cells within gated CD4^+^CD25^+^ cells:** i) Bar diagram showing significant increase in the frequency of Treg cells in the BM-MNCs (disease site) of VL patients (n = 14) than that of PBMCs (*p = 0.001*, *paired t test*). However, the frequency of Teff cells at disease site (BMMNCs) is decreased as compared to peripheral compartment (PBMCs) (Mean±SEM, *p = 0.023, paired t test*). ii) FACS plot shows *in vivo* proliferating (Ki67^+^; an intra nuclear cells proliferating antigen) CD4^+^FoxP3^−^ (Teff) cells in blood and BM of freshly diagnosed VL patients. iii) Significant reduction in the proliferating Teff cells obtained from the disease site of VL patients was observed (*n = 3*; Mean±SD, *p = 0.049, unpaired t test*).

**Figure 2:**
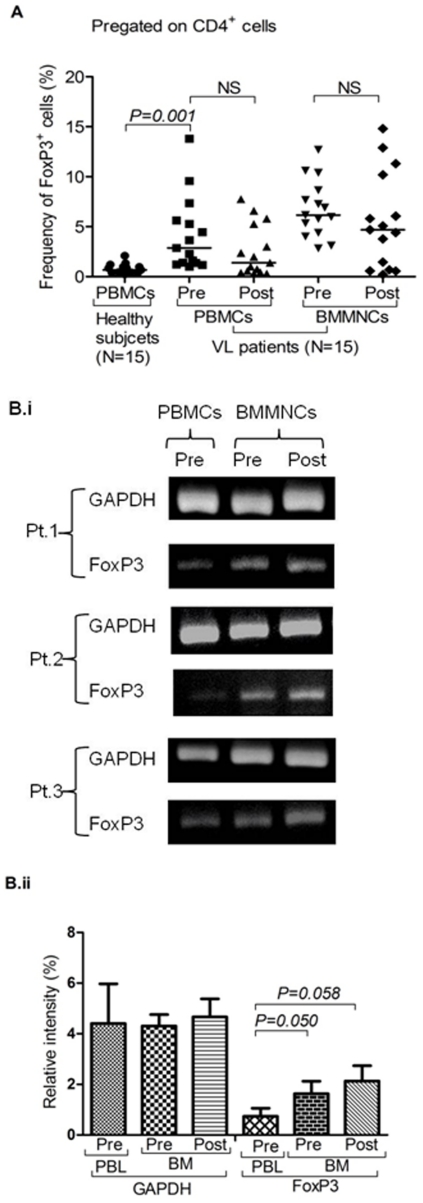
Persistence of Treg cells among visceral leishmaniasis (VL) patients: **A**) **Frequency of CD4^+^ FoxP3^+^ cells after successful therapy:** Scatter plot depicting no statistically significant changes in the frequency of FoxP3^+^ Treg cells after successful therapy (post) in the BM-MNCs and PBMCs of cured VL cases as compared to its pre-treatment levels (pre). Horizontal line in dot plot depicts median value. **B**) **Increased expression of FoxP3 mRNA in BMNCs of patients and their persistence after successful therapy:** (i) Gel photograph showing increased expression of FoxP3 mRNA in BM-MNCs as compared to autologous PBMCs. Persistence of FoxP3 mRNA after successful therapy (post) is also shown in pictures. Individual experiments of three patients with their follow up are shown herewith. (ii) Relative density analysis shows increase in FoxP3 mRNA in patient's BMMNCs at pre (Mean±SD, *p = 0.050, Mann-Whitney test*) and post treatment level (Mean±SD, *p = 0.058, Wilcoxon sign rank test*). However, GAPDH remain unchanged in all three categories.

Our data revealed significant increase of both CD4^+^CD25^+^ cells (representing mixture of activated as well as FoxP3^+^ Treg cells in varying proportions; *p = 0.016, unpaired t test*) and CD4^+^CD25^+^FoxP3^+^ Treg cells (*p = 0.001, paired t test*) in the BM (a major site of parasitic invasion) of the VL patients (*n = 14*) in comparison with autologous peripheral blood (Fig. 1 A, B, C; figure S1, S2). We also observed increase in FoxP3 mRNA in the BMMNCs of the patients to PBMCs using RT PCR (Fig. 2 Bi & B.ii; *p = 0.050, Mann Whitney test*). Compared to the healthy controls (HCs), FoxP3 was more in the PBMCs of VL patients (Fig. 1 D.i & D.ii; *p = 0.018, unpaired t test*). Similar to the observation of Zou et al (2004) [16], we also noted slight Treg cell enrichment in the NBM (*n = 9; Enrichment ratio, ER = 18.28%*). However, their enrichment in the BM of VL patients was of much higher magnitude (Fig. 1C, *p>0.000, unpaired t test*; *ER = 186.93%*), suggesting massive Treg cells enrichment in the BM of VL patients in addition to normal default level of Treg cell trafficking (Fig. 1C). Higher frequency of CD39^+^ Treg (FoxP3^+^) cells in the BM of VL patients (6.5%) compared to that of NBM (0.96%) further substantiates our conclusion (Fig. 1E). Therefore, our data indicates a definitive pathologic enrichment of Treg cells in the BM of VL patients. Enrichment in the BM was observed only for Treg cells (CD4^+^CD25^+^FoxP3^+^; Fig. 1E, *p = 0.001, paired t test*), not for the activated T cells (CD4^+^CD25^+^FoxP3^−^). In fact, the frequency of activated T cells was significantly lower in the BM compared to the blood (Fig. 1E; *p = 0.023, paired t test*). Interestingly, we noted that *ex vivo* proliferation of FoxP3^−^ Teff cells, as identified by Ki67 positivity, was significantly reduced in the BM of VL patients (Fig. 1F.ii, iii & S2; *p = 0.049, unpaired t test*), indicating the strong suppressive influence plausibly induced by Treg cells enrichment at the pathologic site of VL. Overall, our results indicate either i) host's failure to direct the activated effector T cells to at least one major pathologic site of VL or ii) inhibition of host effector T cell activation by the Treg cells recruited at the disease site.

### ii) Persistence of Treg cells among VL patients

The levels of Treg cells failed to reduce significantly, particularly in BM even after successful chemotherapy (Fig. 2A; *n = 15*) and parasite clearance from the BM (Table 1, figure S3). We also observed similar trend in the level of FoxP3 mRNA in BM of VL patients (Fig. 2B i & ii; Table 2). This was quite contrary of our expectation as parasitic clearance fails to reduce or redistribute the Treg cells in the local disease site of VL in human, *just after completion of therapy*. It is also possible that the generation and persistence of FoxP3^+^ Treg cells may not be directly and critically dependent on the parasite load quantitatively, rather their presence and/or persistence may determine the susceptibility *per se* to the development and/or relapse of VL. In fact, we did not find any significant correlation between the parasitic load and the frequency of Treg cells among VL patients (Fig. 1C, Table 1; Correlation coefficient, *r = 0.193, p = 0.510*).

**Table 1:** Details of visceral leishmaniasis patients monitored in the study.

No. of patients	42
**Demographic characteristics**	
Age (Mean ± SD, range)	24.80±16.26 (3–70)
Sex (M/F)	25/17
Ethnicity	Indian
Region	Bihar, India
**Basis of diagnosis**	
**Screening (rK39)**	**No. of cases**
Positive	31
Negative	03
Not tested	08
**Confirmatory (Parasite demonstrations in bone marrow smear, parasite load), pre-therapy**	**No. of cases**
+1	21
+2	14
+3	6
+6	1
**Parasite load, post therapy**	Not detectable (in all cases)
**Other diagnosis**	
WBC count (Mean ± SD, range)	3087.5±1141.8 (1700–5400)
Hb (Mean ± SD, range)	6.81±1.75 (4.3–10.4)
Platelet count (Mean ± SD, range)	97.75±42.88 (21–173)

**Table 2:** Comparative densitometric analysis of the band intensity of FoxP3 mRNA.

Average intensity		
Set-I	Healthy PBMCs	Patients PBMCs
GAPDH	6.43	5.76
FoxP3	0.86	2.133
**Ratio (r)	0.135	0.369
Relative ratio	1	2.7

Set-II	Patients PBMCs	Patients BMMNCsPre treatment	Patients BMMNCsPost treatment
			
GAPDH	4.40	4.30	4.66
FoxP3	0.73	1.63	2.13
**Ratio (r)	0.166	0.379	0.457
Relative ratio	1	2.28	2.75

Ratio of relative intensity shows 2.7 times increase in band intensity of FoxP3 mRNA in patient PBMCs as compared to healthy PBMCs. FoxP3 band intensity is 2.28 times higher among patient's BMMNCs compared to their PBMCs. In cured cases, band intensity is 2.75 times higher than its pre-treatment level. Set I experiments were executed with higher amount of DNA compared to set II, due to extremely lower expression of FoxP3 in healthy subjects. (*Between Patient's PBMCs and BMMNCs at pretreatment level, # Between Patient's BMMNCs at post treatment level, ** r = average intensity of FoxP3 bands/GAPDH bands).

### iii) Antigen induced generation of CD25^+^FoxP3^+^ cells

**Figure 3:**
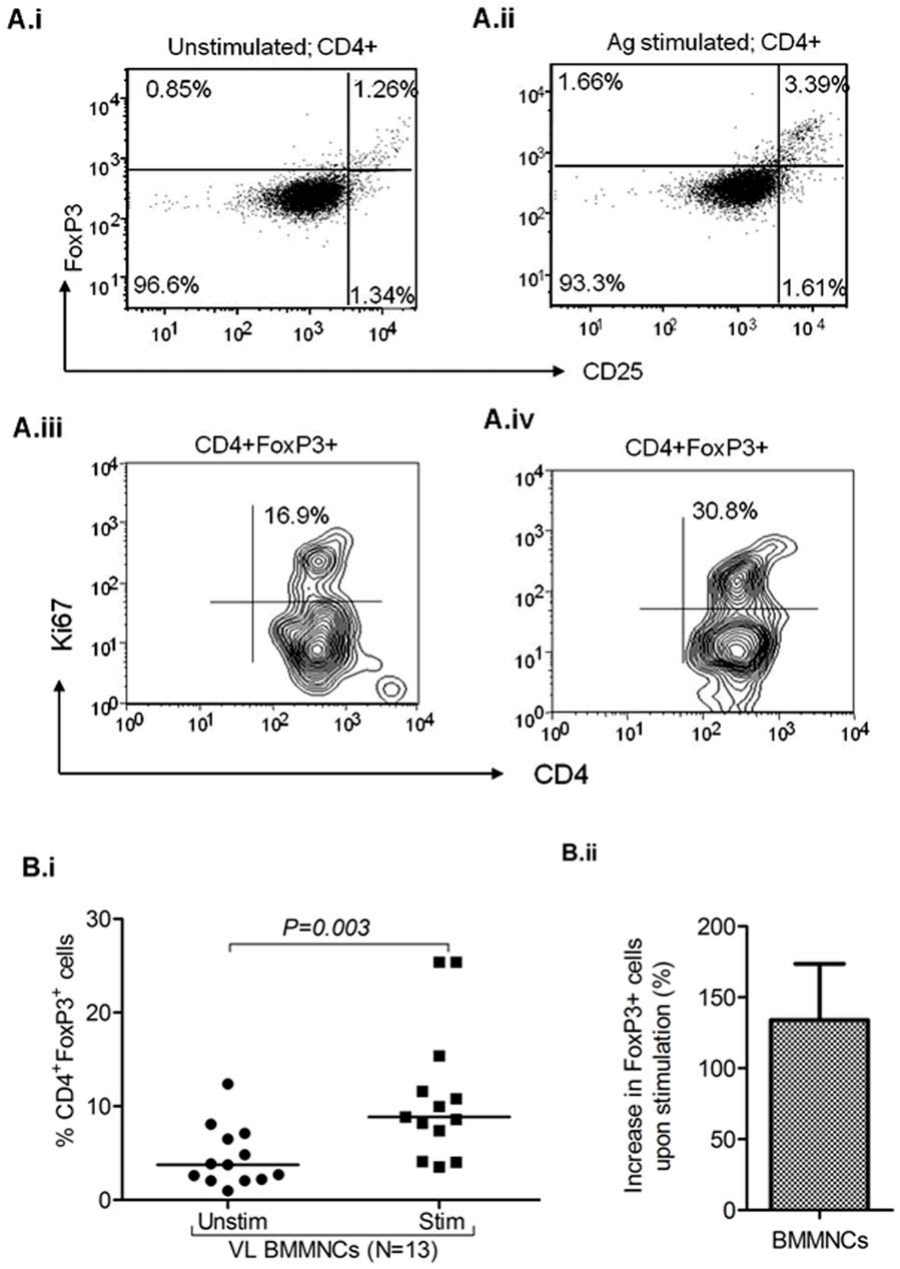
*L. donovani* antigen driven induction of FoxP3^+^ cells in BM-MNCs of visceral leishmaniasis (VL) patients: A i–ii ) Representative plot shows increased frequency of FoxP3^+^ cells (%) in MNCs upon *in vitro* stimulation with *LD* antigen (whole cell lysate, WCL). **A iii–iv**) Representative plot shows increase in the positivity of Ki67 (an intra nuclear cells proliferating antigen) among gated CD4^+^FoxP3^+^ cells upon antigen stimulation (30.8%) as compared to unstimulated cells (16.9%). **B i**) Scatter plot representing the frequency of Foxp3^+^ Treg cells in gated CD4^+^ cells BM-MNCs (*n = 13*) of VL patients upon *in vitro* stimulation with *LD* antigen. Significant increase in the frequency of FoxP3^+^ Treg cells from BM-MNCs of VL patients occur upon antigen stimulation (*p = 0.003, paired t test*). Horizontal line in dot plot depicts median value. **B ii**) Plot shows increase in the frequency of FoxP3^+^ cells in BMMNCs of VL patients upon stimulation (% increase = (frequency of Treg cell in stimulated culture- unstimulated culture)/frequency of Treg in unstimulated×100).

Our data revealed significant increase in the frequency of FoxP3^+^ cells after *in vitro* stimulation with *LD* antigens (whole cell lysate, WCL) in the BM of VL patients (*n = 13*, Fig. 3B.i, *p = 0.003*, *paired t test*). The increase in the frequency of Treg cells upon *in vitro* stimulation (*LD* antigen specific) of BM-MNCs from VL patients raise the possibility of *in vivo* antigen induced generation of Treg cells. Our findings also show marked increase in the frequency of CD4^+^FoxP3^+^ cells co-expressing Ki67 upon *in vitro* stimulation with *LD* antigen (Fig. 3A iii–iv; figure S4). This substantiates our conclusion that at least some fraction of enriched FoxP3^+^ Treg cells is reactive to the leishmanial antigens.

### iv) IL-10 production by the Treg cells derived from VL patients

**Figure 4:**
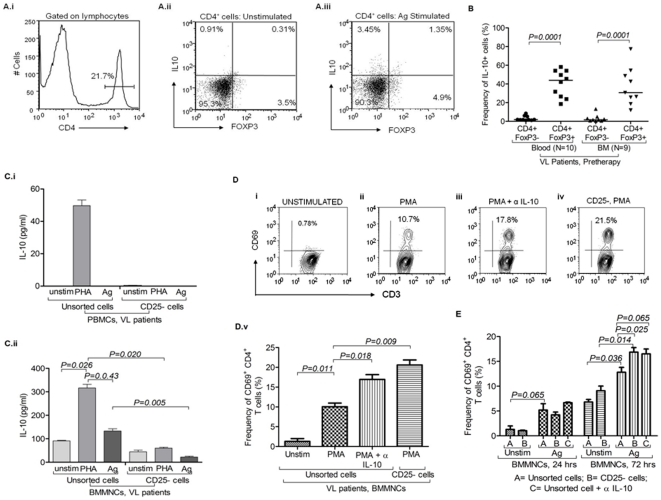
IL-10 production by the Treg cells derived from visceral leishmaniasis (VL) patients: **A**) **IL-10 producing FoxP3^+^ cells in BMMNCs of VLs patients before treatment:** (i) Histogram plot depicts gating strategy of CD4^+^ cells. Bi-variant plots show IL-10 production by CD4^+^FoxP3^+^ Treg cells and CD4^+^FoxP3^−^ cells under different *in vitro* conditions (ii) without stimulation and (iii) with *L. donovani* antigen stimulation. **B**) **IL-10 production by CD4^+^FoxP3^+^ and CD4^+^FoxP3^−^ cells:** Scatter dot plot shows frequency of IL-10 producing cells within gated CD4^+^FoxP3^+^ (Treg) and CD4^+^FoxP3^−^ (Teff) cells in blood as well as in BM of VL patients before anti-*Leishmania* therapy. Data shows that FoxP3^+^ cells are one of the important producers of IL-10 along with CD4^+^FoxP3^−^ cells. Horizontal lines in dot plot depict median value. Significant differences are indicated with *p-values* using paired *t* test. **C**) **CD4^+^CD25^+^ (FoxP3 enriched) cells are major producer of IL-10 at disease site (BMA):** CD25^+^ cells were magnetically sorted out from (**i**) PBMCs and (**ii**) BMMNCs. IL-10 was measured in supernatant of cultured cells (unsorted cells and CD25 depleted cells) stimulated with PHA (mitogen) and *L. donovani* antigen. IL-10 production was dominantly restricted to CD25^+^ cells which were enriched with Treg cells. Data are represented in Mean± SD. Significant differences are indicated with *p-values* using paired *t* test. **D**) **Effect of IL-10 blocking and depletion of FoxP3^+^ enriched cells on T cell activation upon polyclonal stimulation:** **i–v**) *In vitro* PMA stimulation for 24 hrs caused activation of CD4 T cells (CD3^+^CD8^−^) derived from BM-MNCs as measured by the expression of CD69 (% positive cells) on them (v; *p = 0.011*, *paired t test*). Significant increase in the frequency of CD69^+^ early activated CD4 (CD3^+^CD8^−^) T cells occurred upon PMA stimulation of BM-MNCs for 24 hrs when endogenously produced IL-10 was blocked by monoclonal antibody (v; *p = 0.018*, *paired t test*). Similar increase in the frequency of CD69^+^ CD4 (CD3^+^CD8^−^) T cells was also observed when CD4^+^CD25^+^ (Treg enriched) were sorted out using MACS sorting kit and CD25^−^ BMMNCs were cultured with PMA for 24 hrs (v; *p = 0.009, paired t test*). Data are represented in Mean± SD. **E**) **Treg cells and IL-10 suppress** ***L.donovani*** **specific activation of CD4^+^ T cells derived from pathologic site**: *LD* antigen caused significant increase in the frequency of CD69^+^CD4^+^ T cells (*p = 0.036, paired t test*) after 72 hrs of stimulation. Upon blocking of soluble IL-10 by anti IL-10 ab, the frequency of CD69^+^CD4^+^ T cells were increased as compared to antigen alone (*p = 0.065, unpaired t test*). Upon stimulation of CD25 depleted BMMNCs with antigen for 72 hrs, the frequency of CD69^+^CD4^+^ T cells was increased as compared to antigen stimulated unsorted cells (*p = 0.025, paired t test*) as well as unstimulated CD25 depleted cells (*p = 0.014, paired t test*). However, in 24 hrs stimulation experiments, unsorted (with/without blocking of IL-10) and CD25 depleted BMMNCs upon stimulation with *LD* antigen show no significant increase in the frequency of CD69^+^CD4^+^ T cells. {A = unsorted cells (with/without ag stimulation), B = CD25 depleted cells (with/without ag stimulation), C = unsorted cells with IL-10 blocking}. Data are represented in Mean± SD.

CD4^+^FoxP3^+^ Treg cells derived from BM could produce IL-10, whereas, IL-10 production by the CD4^+^FoxP3^−^ T cells was also observed (Fig. 4A & figure S6). Our results suggest that the Treg cell of VL patients, particularly from the BM, responded to *LD* antigen with production of immuno-suppressive cytokine IL-10 (Fig. 4B). Further, we intended to identify the cellular source(s) of IL-10 among VL patients. MNCs derived from the blood and BM of VL patients secreted IL-10 following *in vitro* PHA and antigen stimulation (Fig. 4C). However, the same did not show or showed low levels of IL-10 in PBMCs and BMMNCs respectively, when FoxP3 enriched CD25^+^ cells were depleted by magnetic sorting (as confirmed by FACS based staining for FoxP3 of an aliquot of unsorted MNCs; Fig. 4C i & ii). BM derived MNCs showed spontaneous release of detectable levels of IL-10 *in vitro* (without stimulation), suggesting its presence in higher magnitude in local disease site (Fig. 4C ii). However, Treg enriched CD25^+^ cells may not be the sole source of IL-10 as some levels of IL-10 could still be detected even after depletion of Treg cells (CD25^+^) from the BM MNCs (Fig. 4C.ii). Thus, we conclude that Treg cells are one important source of IL-10 production, particularly in the BM of VL patients and plausibly play critical role in mediating immune-suppression among VL patients.

Blocking of IL-10 along with polyclonal stimulation (Fig. 4D) caused significant increase in the frequency of activated (CD69^+^) CD4 T (CD3^+^CD8^−^) cells as compared to polyclonal/antigenic stimulation alone, respectively. Having shown previously that, among VL patients IL-10 is also produced by the Treg cells, we intended to see the effect of Treg cells depletion on the T cell activation. Frequency of CD69^+^ CD4 T (CD3^+^CD8^−^CD69^+^) cells were increased among CD25 depleted BMMNCs upon polyclonal stimulation (Fig. 4D.iv & v; *p = 0.009, paired t test*). Concentrations of soluble IL-10 were also measured in these experiments (figure S7). With antigen stimulation BMMNCs of VL patients showed significant increase of CD69 expression under both conditions (IL-10 blocking and CD25- depleted cells) as compared to antigen stimulation alone (Fig. 4E; *p = 0.065, unpaired t test & p = 0.025, paired t test* respectively). This indicates immuno-suppressive effect of IL-10 {and its source CD25^+^ (FoxP3^+^) cells} on the antigen specific activation of CD4 T cells. Blocking of IL-10 also increases IFN-γ production upon polyclonal stimulation (figure S8).

### v) Dominance of soluble IL-10, IFN-γ and IL-4 in disease sites

**Figure 5:**
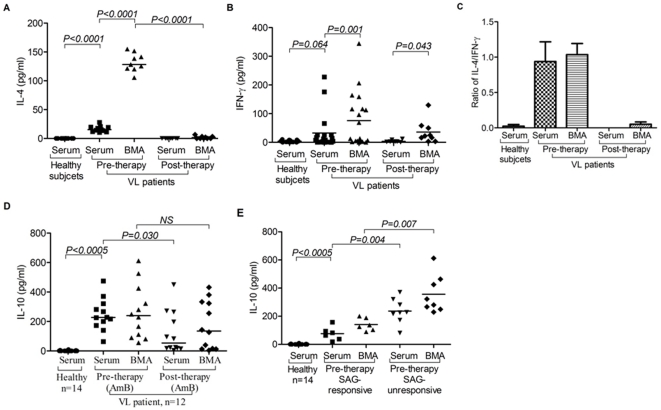
Dominance of soluble suppressive cytokine(s) at disease site (bone marrow, BM) of visceral leishmaniasis (VL) patients: **A**) **High Levels of IL-4 in BM among VL patients:** Scatter plot shows increased level of IL-4 in serum of VL patients (*n = 11*) than that of HCs (*n = 10*; *p = 0.0005, Unpaired t test*). Its level was further increased in BMA of VL patients (*n = 9*; *p = 0.0005*, *Paired t test*). After successful therapy (post), the IL-4 declined to undetectable levels in serum (*n = 8*) and BMA (*n = 9*) of cured VL cases. **B**) **Elevated levels of IFN-γ among VL patients:** Scatter plot depicting higher levels of IFN-γ (pg/ml) in the serum of untreated VL patients (*n = 21*) than that of HCs (*n = 17*; *p = 0.064, unpaired t test*). Level of IFN-γ is further increased in BMA (*n = 19*) as compared to that of autologous serum (*n = 21*, *p = 0.001, Paired t test*). After successful therapy (post), levels of serum IFN-γ is reduced in cured cases (*n = 8*) below their level at 0 day (pre). However, its level in BMA of cured cases (*n = 9*) remained high even after therapy, even though reduced to some extent after successful treatment (*p = 0.043, Paired t test*). **C**) Ratio of IL-4 and IFN-γ shows dominance of IL-4 over IFN-γ in serum as well as BMA before therapy. Post therapy, IFN-γ dominates over IL-4 quantitatively. **D**) **High levels of IL-10 in untreated patients and its persistence after successful therapy with AmB:** Increased levels of IL-10 was observed in the serum as well as BMA of untreated VL patients (*n = 12*) as compared to serum of HCs (*n = 14*; *p = 0.0005, Unpaired t test*). After therapy with AmB, IL-10 levels are maintained at high level in BMA (*n = 12*). **E**) **IL-10 in SAG responsive and unresponsive patients:** Pre treatment levels of IL-10 in the serum and BMA of VL patients could segregate SAG unresponsive patients from the responsive group. IL-10 levels were significantly higher in serum (*p = 0.004*, *Mann Whitney test*) and BMA (*p = 0.007*, *Mann Whitney test*) of patients who did not respond to SAG therapy. Horizontal line in dot plot depicts median value.

We measured levels of pro-inflammatory (IFN-γ) and suppressogenic cytokines (IL-10, IL-4) in serum and BMA (bone marrow aspirate) of VL patients. Our findings demonstrate high levels of IL-10 (Fig. 5D, E.i & E.ii) and IL-4 (Fig. 5A) in serum and BMA of VL patients before treatment. Contrary to our expectation, we observed the persistence of IL-10 in the serum as well as BMA, at least in half of the patients. Interestingly, higher level of IL-4 completely went below the detectable levels after successful therapy. This is suggestive of the fact that IL-4 is more related to the pathologic status of the patients as a signature cytokine, while IL-10 may be more important for immunoregulatory function. Levels of IFN-γ were found to persist even after chemotherapy in the BMA of VL patients, even though its levels declined with treatment (Fig. 5B) indicating that as an effector cytokine, its presence in treatment naïve patients failed to confer protection possibly due to higher pre-therapeutic levels of IL-4 (and IL-10). Thus, decline in the levels of IL-4 may be associated the restoration of immune response necessary for disease containment.

### vi) IL-10 levels and the therapeutic response to SAG among VL patients

Therapeutic response of SAG, a pro-drug requiring its conversion in to active component within activated macrophages, is believed to critically depend on the Th1 effector immune response [4], [5]. We therefore, intended to see if therapeutic response to conventional anti-*Leishmania* drug, SAG is associated with the levels of immune-suppressive cytokine IL-10 or *vice versa*. We measured the levels of soluble IL-10 among untreated VL patients (serum and BMA) and correlated with the clinical outcome of SAG therapy. In a limited number of cases (*n = 15*), we subjected freshly recruited patients to SAG and followed their therapeutic response till the completion of therapy (SAG responsive, *n = 6*; SAG unresponsive cases, *n = 9*). It was interesting to observe a tight inverse correlation between the therapeutic response to SAG and the levels of IL-10 in the serum as well as BMA of VL patients. Taken together, our results demonstrate that SAG failed to cure the cases with higher levels of IL-10, while patients with relatively lower levels of IL-10 responded effectively to SAG with clinical and parasitological cure (Fig. 5E.i).

## Discussion

Recent identification of Treg cells and their dominant suppressive influence on the effector T cell function suggests their critical involvement in VL. Mendez et al. [3] demonstrated that Treg cells predominantly produced IL-10 and mediated the suppression of immune response observed among mice with CL. In an elegant animal study, Suffi et al. [17] showed a definitive role of Treg cells in causing suppression of local effector immune response by altered ratio of Treg and effector T cells and production of IL-10 at the pathologic site. However, the precise cellular origins of IL-10 among VL patients are yet to be deciphered convincingly. Moreover, studies on the Treg cells and their role in suppression of effector T cells response among human VL patients are scanty. Therefore, we attempted to understand the status of Treg cells in terms of their frequency, function and enrichment among VL patients, particularly at the pathologic site(s).

Here, we demonstrate definitive enrichment of Treg cells among VL patients, particularly at major pathologic site (BM). Previously Zou et al reported default Treg cell enrichment in normal bone marrow (NBM) [16]. However, in our study the Treg cell enrichment among VL patients was of profoundly higher magnitude (Fig. 1C), we also noted dominant expression of CCR4 on the robustly enriched Treg cells among VL patients (figure S5). These findings indicate that i) in addition to the normal default enrichment (ER = 18.28%), a massive accumulation of Treg cells occur in the BM of VL patients (ER = 186.93%; *p = 0.001*), ii) plausibly induced by the disease pathology and/parasite burden and iii) their selective recruitment by CCR4 and its ligands. Interestingly, the enriched Treg cells of the BM of VL patients showed evidence for spontaneous *ex vivo* (figure S9) and *in vitro* antigen induced (Fig. 3) proliferation (Ki67 positivity) suggesting their antigen specificity and induction at the pathologic site (BM). We therefore conclude that antigen dependent expansion of Treg cells at the disease milieu contribute to robust Treg cell enrichment observed among VL patients. However, their chemokine receptor driven (CCR4) selective recruitment can not be ruled out and may work synergistically. This is further supported by recent report on generation of suppressive T cells in presence of excess antigenic load [17], [18]. Therefore, enriched Treg cells plausibly suppress effector T cell response to create a state of local immune deficit in human VL. This is confirmed by higher proliferation of peripheral Teff cells relative to the same derived from BM of patients (Fig. 1F ii & iii). Therefore, we conclude that Treg cells play a definitive role in the local suppression of effector T cells. Recent studies [15], [19], using mRNA and FACS based estimation of FoxP3 from the splenic aspirate, did not note Treg cell enrichment at the local disease site of VL patients. In contrast, here we show significant enrichment of CD4^+^CD25^+^FoxP3^+^ (CD39^+^) Treg cells by both mRNA (Fig. 2B) as well as protein level expression (by FACS based staining of the Treg cells with CD4, CD25, FoxP3 and CD39; Fig. 1C & E) in the BM of the patients with VL. The discrepancy between the findings of the present study and that of Nylen et al [15] may be due to the differences in the local disease sites (spleen and BM) and/or the dynamics of Treg cell enrichment in organ specific manner.

Previous studies on animal models of protozoan infections including CL [13], [14] and among VL patients [15] demonstrated higher levels of IL-10 and identified FoxP3- T cells as its source. However, we observed that the CD4^+^FoxP3^+^ Treg cells produced IL-10 in an antigen dependent manner along with CD4^+^FoxP3- cells (Fig. 4A & B). Reduction in the IL-10 production after depletion of CD25^+^ (FoxP3^+^ enriched) cells substantiates our conclusion that FoxP3+ Treg cells are important source of IL-10 among VL patients (Fig. 4C). Moreover, increase in the frequency of *LD* specific CD69^+^ activated T cells upon blocking IL-10 in our hands strongly suggests IL-10 mediated suppression of T cell activation among VL patients (Fig. 4E) Taken together, here we provide convincing evidences for i) antigen induced expansion and enrichment Treg cells in at the pathologic site (BM), ii) cellular source (FoxP3^+^ Treg and FoxP3- non-Treg cells) of IL-10 and iii) IL-10 mediated suppression of *LD* specific effector T cell response in human VL. Thus, inhibiting the recruitment, expansion and/or the function (particularly IL-10 production) of Treg cells may constitute a major approach for restoration of the local effector immune response among VL patients.

Considering the suppressive role of Treg cells and IL-10, successful chemotherapy is envisaged to reverse the suppressed state of immunity. Contrary to our expectation and existing literature [15], [19], persistence of Treg cells (Fig. 2A) and IL-10 (Fig. 5D) were observed even after the successful chemotherapeutic among half of the cured VL cases. Study of Murray et al [20] on achieving sterile cure with blocking IL-10 pathway in mouse VL model relates to the findings of our study. Persistent presence of Treg cells and their selective recruitment at the disease site(s) may play critical role in persistence of residual parasite burden even after clinically effective chemotherapy, which may result in the relapse of the disease, development of PKDL [21] and/or susceptibility to re-infection. Our results provide mechanistic insight in to its mechanism parasite persistence. Interestingly, the levels of Th2 associated cytokine IL-4 decreased to the undetectable level (along with elevated levels of IFN-γ) following chemotherapy (Fig. 5A, B). Taken together, this suggests that subsequent to effective chemotherapy, a fine balance ensues between the protective cytokine IFN-γ and immuno-suppressive cytokine IL-10 (plausibly derived from the persisting Treg cells) and tilting its balance towards the later at any subsequent point of time may cause disease relapse [9]. Delineating the factors triggering such events is important for understanding the immunopathology of relapse of VL and occurrence of PKDL in human.

Chemotherapy of VL with first line of relatively inexpensive drug, SAG has suffered a major setback due to development of unresponsiveness [22], [23]. This has compounded the VL elimination program among impoverished population inflicted with this disease in India and adjoining region [22]. SAG is a pentavalent antimonial pro-drug requiring its conversion in the host into its active trivalent form [4], [24]. Conversion of SAG primarily occurs by reactive oxygen species (ROI) following Th1 response (IFN-γ) dependent activation of macrophages [4], [24]. Thus, synergistic and complementary function of the chemotherapy and the protective immunity constitute the drug immune interphase and believed to determine the efficacy of SAG. We hypothesized that IL-10 mediated suppression of immunity plausibly dictate the response to SAG therapy among VL patients. We noted a tight and inverse correlation between the levels of IL-10 and response to SAG therapy (Fig. 5E). We propose that elevated levels of IL-10 released primarily from the enriched Treg cells shift the dynamics of drug immune interphase towards immune-suppression, thus preventing the induction of proinflammatory cytokines (IFN-γ), macrophage activation and lead to SAG unresponsiveness. Reversing the Treg cell recruitment and inhibiting their IL-10 production or blocking the IL-10 at the pathologic site(s) may constitute an effective intervention modality for repairing the immune deficit of VL patients and thereby contain the infection as well as improve the drug (SAG) response. Moreover, levels of IL-10 and Treg cells in the BM may prove to be a potent bio-marker of the disease severity and drug response of VL patients.

## Materials and Methods

### 1) Healthy subjects and patients

The study included 42 parasitologically confirmed patients [mean age 24.80±16.26 yrs; range 3–70 yrs; 25 males and 17 females). These patients attended Balaji Utthan Sansthan (BUS), Patna, Bihar, India for diagnosis and treatment. Quantification of parasite before and after therapy was performed in the BM of the recruited patients [25]. Seventeen HCs {Healthy family members and non family members (living in the same endemic region)}, chosen from among the persons attending patients, were also included in the study. All subjects were HIV negative. None of the patients was on anti-*Leishmania* treatment at the time of enrolment in the study. Normal bone marrow (NBM) specimens were obtained from attendees at our haematology clinic for diagnosis of presumptive hematologic disorders (such as idiopathic thrombocytopenic purpura (ITP), hypersplenomegaly, anaemia etc.) Their bone marrow aspiration was performed as a part of routine diagnostic evaluation. We recruited 11 such cases with patient's informed consent. Subsequently, 9 cases were confirmed normal clinically as well as haematologically and recruited in the study.

Samples (Blood, BM and serum) from these patients were collected in heparinized tubes (BD Vacutainer™ sodium heparin, Cat. No. 366480, Becton Dickinson, Franklin Lakes, NJ) and transported to All India Institute of Medical Sciences (AIIMS), New Delhi. BMA were collected for diagnostic confirmation (demonstration of parasite) (Table 1). Patients were kept on continuous monitoring in hospitals.

### 2) Therapy

Recruited patients were treated with SAG (20 mg/kg body weight for 28 days) and AmphotericinB (AmB; 1 mg/kg body weight for 20 days) as per WHO guidelines. Treatment was stopped for patients who did not respond to SAG and/or developed cardiac illness and after interval of 1 or 2 days patients were instituted on therapy with a second line of drug like AmB.

### 3) Ethics Statement

The research project was approved by the Institutional Ethics Committee of AIIMS, New Delhi (Ref. No. B-11/6.10.2006; October 17, 2006) and BUS, Patna, Bihar. Written informed consent was obtained from all study participants including healthy subjects. For minor children and for patients those who cannot read and write, written informed consent was provided by their legal guardian.

### 4) Reagents

RPMI medium (Roswell Park Memorial Institute; Caisson Laboratories Inc., Cat: 010P, with L-glutamine, without HEPES and NaHCO_3_) supplemented with L-glutamine (G-5763, Sigma Chemicals Co., St. Louis, USA), antibiotics (Pen-Strep-Ampho Sol; Biological Industries, Kibbutz Beit Haemek, Israel) and fetal calf serum (10%, Biological Industries, Kibbutz Beit Haemek, Israel) were used for cell culture experiments. Permeablization of cells for intracellular antigen detection was done with 0.3% Saponin (S-7900, Sigma Chemicals Co., St. Louis, USA). Monoclonal antibodies used were FITC conjugated CD3, CD4, CD39, IFN-γ (BD Pharmingen, San Diego, CA, USA); PE conjugated CD3, CD25, CD69, Ki67 set (all BD pharmingen, San Diego, CA, USA); PerCP/Cy5PE conjugated CD4, CD8 (all BD Pharmingen, San Diego, CA, USA); APC conjugated CD3, CD4 (all BD Pharmingen, San Diego, CA, USA). FoxP3 staining kits were purchased from e-Biosciences (Cat. No. 71-5776-40, e-Biosciences, San Diego, CA, USA). α-CD25 microbead antibodies along with column (MACS^R^ MS separation column) and magnetic sorting apparatus were purchased from Miltenyi Biotec.

### 5) Isolation of MNCs from blood and BMA

Briefly, mononuclear cells (MNCs) were isolated from heparinized blood and BMA by Ficoll Hypaque gradient centrifugation, washed thrice with incomplete RPMI (without FCS) and finally suspended in complete RPMI-1640 supplemented with 10% FCS (Caisson Laboratories, Logan, UT). The viability of cells was checked by trypan blue dye exclusion test and was more than 98% [12]. Before the density gradient centrifugation, the BMA was centrifuged at 2000 rpm for 5 mins in micro-centrifuge. The pellet containing cells will be separated from the liquid part, which will be used for the detection of soluble cytokine(s) in BMA.

### 6) Surface and intracellular staining and *in vitro* culture of cells

Briefly, isolated cells were incubated directly with fluorescence labeled monoclonal antibodies in staining buffer (PBS+BSA+azide) for 15 min on ice. After washing, fixation and re-washing, cells were transferred in FACS tube (BD FALCON) for data acquisition by three/four-color flow cytometer (Beckton Dickinson, FACS calibur). For the staining of intra-cytoplasmic antigens (e.g. IL-10, Ki67 etc), cells were permeabilized by using permeabilization buffer (0.3% saponin) after fixation for 30 mins at room temperature [12].

Cells were cultured (2×10^6^cells/ml) in microtitre plate (96 well and U bottom plates, BD Falcon) in presence of PHA (5 ng/ml, Sigma-aldrich co, St. Louis, USA) for 72 hours at 5% CO_2_ at 37°C. For antigen stimulation study, cells were incubated with *LD* antigen (whole cell lysate; 10 µg/ml) with purified α-CD28 and α-CD49d antibody (AbD Sertec, Oxford, UK, LE/AF, Cat. No. MCA70EL & MCA923EL respectively) for 24 hrs and monensin (Golgi transport inhibitor; 1 µM, Sigma-aldrich co, St. Louis, USA) was added in last 6 hrs (20). For Blocking studies, unconjugated anti-IL-10 monoclonal antibody (NA/LE, BD Pharmingen, San diego, CA, USA) was mixed with MNCs (2 million/ml) prior to the addition of PHA [12].

### 7) Staining of cells for FoxP3 antigen

Briefly, 1–2×10^6^ MNCs were first, surface stained (e.g. CD4, CD25, CD39 and CCR4 etc) and were stained for FoxP3 antigen using FoxP3 staining (FITC/PE) kit (Cat. No. 00-5123-43, ebiosciences, San diego, CA, USA). To assure specificity of CD4, CD25 & FoxP3 staining, we performed fluorescence minus one (FMO) and isotype staining (figure S1) of the same specimens [12]. To evaluate the proliferation of Treg and Teff cells, cells were stained with anti-Ki67 and anti-FoxP3 abs following *in vitro* stimulation with *LD* antigens using FoxP3 staining protocol (figure S4). To enumerate the IL-10 producing FoxP3^+^ cells (figure S6), antigen stimulated cells were stained for IL-10 and FoxP3 using the same protocol. To confirm the staining specificity, we performed fluorescence minus one (FMO) for IL-10 (figure S6). For enumeration of FoxP3^+^ Treg cells, 2–5×10^6^ events (depending on cell yield) were acquired to obtain analyzable number of FoxP3^+^ cells.

### 8) Amplification of FoxP3 mRNA

Total mRNA was extracted from the MNCs obtained from peripheral blood and local disease site specimen, using Trizol reagent (Sigma-Aldrich Inc., St. Louis, USA) as recommended by the manufacturer. mRNA was converted into cDNA by Reverse Transcriptase -PCR. Quality was assessed using ND-1000 spectrophotometer (NanoDrop Technologies, USA). Isolated, precipitated & quantified cDNA was then utilized for the amplification of Foxp3. GAPDH (housekeeping gene) was used as a positive control. All constituents of the PCR mix except the individual cDNA samples were mixed together and mixed thoroughly. 21 µl of the reaction mix was dispensed into PCR tubes and 0.5–1 µg (5 µl) individual cDNA was added to each tube. Sterile distilled H_2_O and standard cDNA were used as negative and positive controls, respectively. The tubes were spun down briefly for 10–15 seconds and placed on the heat block of thermal cycler. The amplification was accomplished on a DNA thermal cycler (GeneAmp PCR System 9700, Applied Biosystems, USA) at successive incubation steps at three different temperatures as follows. Initial incubation at 94°C - 5 min followed by 94°C – 1 min (denaturation), 57.7°C -1 min (annealing), 72°C – 1.5 min (amplification) for 35 cycles.

Following sets of primers were used:

FoxP3 F 5′ TGC CTC CTC TTC TTC CTT GA 3′

 R 5′ CCA CTT GCA GAC ACC ATT TG 3′

GAPDH F 5′ AAA ATC AAG TGG GGC GAT GC 3′

 R 5′ TGA GCT TGA CAA AGT GGT CG 3′

1.5% agarose gel was used to analyze PCR bands. A molecular weight DNA marker (Bangalore GENEI, India) having uniform banding pattern of 100 bp to 1000 bp was loaded in the lateral well. DNA bands of 439 bp were identified relative to the markers used and photographed using Computer Aided Gel documentation system (Syngene, Cambridge, UK) [12].

### 9) ELISA for IL-10, IL-4 and IFN-γ

Sandwich ELISA for soluble cytokines (IL-4: Cat. No. 555142; IL-10: Cat. No. 555157 & IFN-γ: Cat. No. 555142; BD Pharmingen, San diego, CA, USA) and chemokines (MDC: Cat. No. DMDOO & TARC: Cat. No. DDNOO; Quantikine immunoassay, R&D system, Minneapdis, MN, USA) were performed in serum and BMA of patients. After development of color, stop solutions were added to stop the reaction and O.D. was taken at 590 nm in a 96 well plate ELISA reader (Anthos 2020; Anthos Labtec Instruments).

### 10) Culture of parasite *Leishmania donovani*

Clinical isolate of promastigotes (from Balaji Utthan Sansthan, Bihar) were maintained in modified DMEM supplemented with 10% FCS, adenosine, hemin, biotin and triethanolamine etc., at 25°C in BOD incubator. These were maintained in the laboratory by frequent passaging of cells, until the colour of the medium became yellowish and/or cells became confluent.

### 11) Preparation of antigen of *L. donovani*

Parasites from log phase growth were collected by centrifugation (×500 g) for 10 min at 4°C. Pellet of *LD* was washed twice by PBS and resuspended in sterile PBS and was sonicated in BRANSON 250 sonicator, using 50% duty cycle (i.e. 30 seconds on, 30 seconds off) for 15 mins at 4°C. Sonicate was mixed and the protein concentration was assessed with a ND-1000 spectrophotometer (NanoDrop Technologies, USA). 10 µg of protein was used to stimulate 1–2 million of cells in one ml [26].

### 12) Statistical analysis

The normal continuous variables are presented in mean and with standard deviation. However, non-normal co-variants are shown in the median and interquartile range. For the comparison of all continuous variables between the studied group, paired t-test/Wilcoxon Sign rank test (normal/non-normal) and unpaired t test/Mann Whitney test (normal/non-normal) are used. P-value less than 5% level of significance will regarded as significant results. Horizontal line in the scatter plot represents the median value and bar diagram shows mean±SD of data set. The analysis is done using SPSS 15.0. Graphs and figures were made in GraphPad Prism 5 version.

## Supporting Information

Figure S1 **FACS contour plot showing gating strategy for CD4^+^CD25^+^FoxP3^+^ Treg cells.** **i**) Bi-variant contour plot showing co-expression of CD25 and FoxP3 on gated CD4^+^ T cells in BMMNCs of VL patients. **ii**) Representative FACS contour plot depicting the gating strategy to enumerate CD4^+^CD25^+^FoxP3^+^ Treg cells among VL patients. 2–5×10^6^ events were acquired to achieve sufficiently sizable number of FoxP3^+^ for further analysis. Gating of positive (vs negative) cells was based on either isotype and/or fluorescence minus one (FMO) staining. Upper panel shows plots demonstrating isotype control for CD25 staining respectively. FoxP3^+^ cells were enumerated on gated CD4^+^CD25^+^ cells using isotype control for FoxP3 staining (lower panel).
(DOC)

Figure S2 **Level of FoxP3 in CD4+CD25- cells from visceral leishmaniasis (VL) patients:** Bar diagram shows frequency of FoxP3^+^ cells among CD4^+^CD25^+^ and CD4^+^CD25^−^ cells from blood and bone marrow (BM) of VL patients (n = 14).
(DOC)

Figure S3 **Clearance of parasite from BM after completion of therapy:** Representative image photographs of Giemsa stained BM smear slides of VL patients (*n = 5*) showing presence and disappearance of *LD* in the BM of VL patients before and after anti-*Leishmania* therapy respectively. Inset shows magnified view of *LD* bodies.
(DOC)

Figure S4 **Staining of Ki67^+^ proliferating CD4 cells:** For *in vitro* antigen induced or *in vivo* proliferation assay of Treg cells, mononuclear cells were stained for Ki67 (an intra-nuclear cell proliferating antigen) and FoxP3 using FoxP3 staining protocol. To confirm our staining, isotype staining was also performed for Ki67. Contour plots show isotype and Ki67 staining of gated CD4^+^FoxP3^−^ cells derived from PBMCs of VL patients.
(DOC)

Figure S5 **Higher frequency of CCR4^+^ Treg cells (FoxP3^+^) in BM of VL patient as opposed to NBM:** **i**) On gated CD4^+^ T cells, co-expression of CCR4 and FoxP3 was observed. **ii**) Expression of CCR4 was observed on gated CD4^+^FoxP3^+^ and CD4^+^FoxP3^−^ cells. Overlay histogram shows increased frequency of CCR4^+^ cells among gated Treg population (thin line) as compared to CD4^+^FoxP3^−^ population (solid line) of BMMNCs of VL patients. iii) Overlay histogram shows increased frequency of CCR4^+^ cells among gated Treg population (thin line) as compared to CD4^+^FoxP3^−^ population (solid line) of normal BMMNCs. iv) Scatter plot shows increased number of CCR4^+^ Treg cells among VL patients.
(DOC)

Figure S6 **Treg cells are one of the producers of IL-10 in visceral leishmaniasis (VL) patients:** (**i–iii**) FACS contour plots show co-expression of FoxP3 and IL-10 PBMCs of healthy subject upon (iii) polyclonal stimulation as compared to (i) unstimulated. (ii) Gating is based on the fluorescence minus one (FMO) staining for IL-10. (**iv–v**) FACS dot plot shows IL-10 production by FoxP3^+^ and FoxP3^−^ cells among CD4 T cells derived from blood of VL patients under different *in vitro* conditions (iv) no stimulation and (v) *L. donovani* antigen stimulation. (**vi–vii**) Data shows that FoxP3^+^ cells from bone marrow (BM) are one of the important producers of IL-10 along with CD4^+^FoxP3^−^ cells upon *in vitro* stimulation with *L. donovani antigen*.
(DOC)

Figure S7 **Activation of T cells is repealed upon sorting out CD25+ cells and IL-10 blocking:** (A) Expression of CD69 is measured to estimate activation of T cells. Upon mitogenic stimulation (PHA), cells from bone marrow of VL patients are significantly expressing CD69, which is further increased if CD25 depleted cells are stimulated with PHA. The similar effect is observed when soluble IL-10 is blocked with anti-IL-10 monoclonal ab. (B) Culture supernatant from experiment-A was stored and estimated for soluble IL-10. Finding suggests that IL-10 was significantly decreased upon sorting out CD25+ cells, indicating that the one of the cellular source of IL-10 is CD25+ cells.
(DOC)

Figure S8 **Immuno-regulatory IL-10 suppresses the IFN-γ production upon polyclonal stimulation:** **i–iv**) *In vitro* PMA stimulation of BM-MNCs from VL patients (n = 5) caused drastic production of IFN-γ by CD8^+^T cells (iv; *p = 0.029, unpaired t test*). Significant increase in the production of IFN-γ by CD8^+^T cells upon PMA stimulation of BM-MNCs for 24 hrs when endogenously produced IL-10 was blocked by monoclonal antibody (iii & iv; *p = 0.032, unpaired t test*).
(DOC)

Figure S9 **Proliferation of non-Treg cells are inversely correlated with the frequency of FoxP3^+^ Treg cells in bone marrow (BM) of VL patients:** Data shows analysis of proliferating Treg (Ki67^+^FoxP3^+^) and non-Treg (Ki67^+^FoxP3^−^) cells from (**i & ii**) blood and (**iii & iv**) BM of VL patients. Findings show decreased proliferation of CD4+FoxP3- cells at the disease sites (BM) compared to blood and sign of spontaneous proliferation of FoxP3^+^ Treg cells Gating is based on the isotype staining for Ki67 (i & iii).
(DOC)
