# Supplementary material for: Regulatory T Cells Suppress T Cell Activation at the Pathologic Site of Human Visceral Leishmaniasis
Source: PLoS One. 2012 Feb 8;7(2):e31551. doi: 10.1371/journal.pone.0031551 (PMC3275558; doi:10.1371/journal.pone.0031551)
Supplement: Figure S1 — FACS contour plot showing gating strategy for CD4+CD25+FoxP3+ Treg cells. i) Bi-variant contour plot showing co-expression of CD25 and FoxP3 on gated CD4+ T cells in BMMNCs of VL patients. ii) Representative FACS contour plot depicting the gating strategy to enumerate CD4+CD25+FoxP3+ Treg cells among VL patients. 2–5×10^6^ events were acquired to achieve sufficiently sizable number of FoxP3+ for further analysis. Gating of positive (vs negative) cells was based on either isotype and/or fluorescence minus one (FMO) staining. Upper panel shows plots demonstrating isotype control for CD25 staining respectively. FoxP3+ cells were enumerated on gated CD4+CD25+ cells using isotype control for FoxP3 staining (lower panel). (DOC) [file pone.0031551.s001.doc]

**Figure S1**

**Figure S1: FACS contour plot showing gating strategy for CD4^+^CD25^+^FoxP3^+^ Treg cells. i)** Bi-variant contour plot showing co-expression of CD25 and FoxP3 on gated CD4^+^ T cells in BMMNCs of VL patients. **ii)** Representative FACS contour plot depicting the gating strategy to enumerate CD4^+^CD25^+^FoxP3^+^ Treg cells among VL patients. 2-5x10^6^ events were acquired to achieve sufficiently sizable number of FoxP3^+^ for further analysis. Gating of positive (vs negative) cells was based on either isotype and/or fluorescence minus one (FMO) staining. Upper panel shows plots demonstrating isotype control for CD25 staining respectively. FoxP3^+^ cells were enumerated on gated CD4^+^CD25^+^ cells using isotype control for FoxP3 staining (lower panel).
