# Supplementary material for: Regulatory T Cells Suppress T Cell Activation at the Pathologic Site of Human Visceral Leishmaniasis
Source: PLoS One. 2012 Feb 8;7(2):e31551. doi: 10.1371/journal.pone.0031551 (PMC3275558; doi:10.1371/journal.pone.0031551)
Supplement: Figure S2 — Level of FoxP3 in CD4+CD25- cells from visceral leishmaniasis (VL) patients: Bar diagram shows frequency of FoxP3+ cells among CD4+CD25+ and CD4+CD25− cells from blood and bone marrow (BM) of VL patients (n = 14). (DOC) [file pone.0031551.s002.doc]

**Figure S2**

**Figure S2. Level of FoxP3 in CD4+CD25- cells from visceral leishmaniasis (VL) patients:** Bar diagram shows frequency of FoxP3^+^ cells among CD4^+^CD25^+^ and CD4^+^CD25^-^ cells from blood and bone marrow (BM) of VL patients (n=14).
