# Supplementary material for: Regulatory T Cells Suppress T Cell Activation at the Pathologic Site of Human Visceral Leishmaniasis
Source: PLoS One. 2012 Feb 8;7(2):e31551. doi: 10.1371/journal.pone.0031551 (PMC3275558; doi:10.1371/journal.pone.0031551)
Supplement: Figure S3 — Clearance of parasite from BM after completion of therapy: Representative image photographs of Giemsa stained BM smear slides of VL patients (n = 5) showing presence and disappearance of LD in the BM of VL patients before and after anti-Leishmania therapy respectively. Inset shows magnified view of LD bodies. (DOC) [file pone.0031551.s003.doc]

**Figure S3**

**Figure S3. Clearance of parasite from BM after completion of therapy:** Representative image photographs of Giemsa stained BM smear slides of VL patients (*n=5*) showing presence and disappearance of *LD* in the BM of VL patients before and after anti-*Leishmania* therapy respectively. Inset shows magnified view of *LD* bodies.
