# Supplementary material for: Regulatory T Cells Suppress T Cell Activation at the Pathologic Site of Human Visceral Leishmaniasis
Source: PLoS One. 2012 Feb 8;7(2):e31551. doi: 10.1371/journal.pone.0031551 (PMC3275558; doi:10.1371/journal.pone.0031551)
Supplement: Figure S4 — Staining of Ki67+ proliferating CD4 cells: For in vitro antigen induced or in vivo proliferation assay of Treg cells, mononuclear cells were stained for Ki67 (an intra-nuclear cell proliferating antigen) and FoxP3 using FoxP3 staining protocol. To confirm our staining, isotype staining was also performed for Ki67. Contour plots show isotype and Ki67 staining of gated CD4+FoxP3− cells derived from PBMCs of VL patients. (DOC) [file pone.0031551.s004.doc]

**Figure S4**

**Figure S4: Staining of Ki67^+^ proliferating CD4 cells:** For *in vitro* antigen induced or *in vivo* proliferation assay of Treg cells, mononuclear cells were stained for Ki67 (an intra-nuclear cell proliferating antigen) and FoxP3 using FoxP3 staining protocol. To confirm our staining, isotype staining was also performed for Ki67. Contour plots show isotype and Ki67 staining of gated CD4^+^FoxP3^-^ cells derived from PBMCs of VL patients.
