# Supplementary material for: Regulatory T Cells Suppress T Cell Activation at the Pathologic Site of Human Visceral Leishmaniasis
Source: PLoS One. 2012 Feb 8;7(2):e31551. doi: 10.1371/journal.pone.0031551 (PMC3275558; doi:10.1371/journal.pone.0031551)
Supplement: Figure S5 — Higher frequency of CCR4+ Treg cells (FoxP3+) in BM of VL patient as opposed to NBM: i) On gated CD4+ T cells, co-expression of CCR4 and FoxP3 was observed. ii) Expression of CCR4 was observed on gated CD4+FoxP3+ and CD4+FoxP3− cells. Overlay histogram shows increased frequency of CCR4+ cells among gated Treg population (thin line) as compared to CD4+FoxP3− population (solid line) of BMMNCs of VL patients. iii) Overlay histogram shows increased frequency of CCR4+ cells among gated Treg population (thin line) as compared to CD4+FoxP3− population (solid line) of normal BMMNCs. iv) Scatter plot shows increased number of CCR4+ Treg cells among VL patients. (DOC) [file pone.0031551.s005.doc]

**Figure S5**

**Figure S5: Higher frequency of CCR4^+^ Treg cells ( FoxP3^+^) in BM of VL patient as opposed to NBM: i)** On gated CD4^+^ T cells, co-expression of CCR4 and FoxP3 was observed. **ii)** Expression of CCR4 was observed on gated CD4^+^FoxP3^+^ and CD4^+^FoxP3^-^ cells. Overlay histogram shows increased frequency of CCR4^+^ cells among gated Treg population (thin line) as compared to CD4^+^FoxP3^-^ population (solid line) of BMMNCs of VL patients. iii) Overlay histogram shows increased frequency of CCR4^+^ cells among gated Treg population (thin line) as compared to CD4^+^FoxP3^-^ population (solid line) of normal BMMNCs. iv) Scatter plot shows increased number of CCR4^+^ Treg cells among VL patients.
