# Supplementary material for: Regulatory T Cells Suppress T Cell Activation at the Pathologic Site of Human Visceral Leishmaniasis
Source: PLoS One. 2012 Feb 8;7(2):e31551. doi: 10.1371/journal.pone.0031551 (PMC3275558; doi:10.1371/journal.pone.0031551)
Supplement: Figure S6 — Treg cells are one of the producers of IL-10 in visceral leishmaniasis (VL) patients: (i–iii) FACS contour plots show co-expression of FoxP3 and IL-10 PBMCs of healthy subject upon (iii) polyclonal stimulation as compared to (i) unstimulated. (ii) Gating is based on the fluorescence minus one (FMO) staining for IL-10. (iv–v) FACS dot plot shows IL-10 production by FoxP3+ and FoxP3− cells among CD4 T cells derived from blood of VL patients under different in vitro conditions (iv) no stimulation and (v) L. donovani antigen stimulation. (vi–vii) Data shows that FoxP3+ cells from bone marrow (BM) are one of the important producers of IL-10 along with CD4+FoxP3− cells upon in vitro stimulation with L. donovani antigen. (DOC) [file pone.0031551.s006.doc]

**Figure S6**

**Figure S6:** **Treg cells are one of the producers of IL-10 in visceral leishmaniasis (VL) patients:** **(i-iii)** FACS contour plots show co-expression of FoxP3 and IL-10 PBMCs of healthy subject upon (iii) polyclonal stimulation as compared to (i) unstimulated. (ii) Gating is based on the fluorescence minus one (FMO) staining for IL-10. **(iv-v)** FACS dot plot shows IL-10 production by FoxP3^+^ and FoxP3^-^ cells among CD4 T cells derived from blood of VL patients under different *in vitro* conditions (iv) no stimulation and (v) *L. donovani* antigen stimulation. (**vi-vii)** Data shows that FoxP3^+^ cells from bone marrow (BM) are one of the important producers of IL-10 along with CD4^+^FoxP3^-^ cells upon *in vitro* stimulation with *L. donovani antigen*.
