# Supplementary material for: Regulatory T Cells Suppress T Cell Activation at the Pathologic Site of Human Visceral Leishmaniasis
Source: PLoS One. 2012 Feb 8;7(2):e31551. doi: 10.1371/journal.pone.0031551 (PMC3275558; doi:10.1371/journal.pone.0031551)
Supplement: Figure S7 — Activation of T cells is repealed upon sorting out CD25+ cells and IL-10 blocking: (A) Expression of CD69 is measured to estimate activation of T cells. Upon mitogenic stimulation (PHA), cells from bone marrow of VL patients are significantly expressing CD69, which is further increased if CD25 depleted cells are stimulated with PHA. The similar effect is observed when soluble IL-10 is blocked with anti-IL-10 monoclonal ab. (B) Culture supernatant from experiment-A was stored and estimated for soluble IL-10. Finding suggests that IL-10 was significantly decreased upon sorting out CD25+ cells, indicating that the one of the cellular source of IL-10 is CD25+ cells. (DOC) [file pone.0031551.s007.doc]

**Figure S7**

**Figure S7: Activation of T cells is repealed upon sorting out CD25+ cells and IL-10 blocking:** (A) Expression of CD69 is measured to estimate activation of T cells. Upon mitogenic stimulation (PHA), cells from bone marrow of VL patients are significantly expressing CD69, which is further increased if CD25 depleted cells are stimulated with PHA. The similar effect is observed when soluble IL-10 is blocked with anti-IL-10 monoclonal ab. (B) Culture supernatant from experiment-A was stored and estimated for soluble IL-10. Finding suggests that IL-10 was significantly decreased upon sorting out CD25+ cells, indicating that the one of the cellular source of IL-10 is CD25+ cells.
