# Supplementary material for: Regulatory T Cells Suppress T Cell Activation at the Pathologic Site of Human Visceral Leishmaniasis
Source: PLoS One. 2012 Feb 8;7(2):e31551. doi: 10.1371/journal.pone.0031551 (PMC3275558; doi:10.1371/journal.pone.0031551)
Supplement: Figure S8 — Immuno-regulatory IL-10 suppresses the IFN-γ production upon polyclonal stimulation: i–iv) In vitro PMA stimulation of BM-MNCs from VL patients (n = 5) caused drastic production of IFN-γ by CD8+T cells (iv; p = 0.029, unpaired t test). Significant increase in the production of IFN-γ by CD8+T cells upon PMA stimulation of BM-MNCs for 24 hrs when endogenously produced IL-10 was blocked by monoclonal antibody (iii & iv; p = 0.032, unpaired t test). (DOC) [file pone.0031551.s008.doc]

**Figure S8**

**Figure S8: Immuno-regulatory IL-10 suppresses the IFN-γ production upon polyclonal stimulation:** **i-iv)** *In vitro* PMA stimulation of BM-MNCs from VL patients (n=5) caused drastic production of IFN-γ by CD8^+^T cells (iv; *p= 0.029, unpaired t test*). Significant increase in the production of IFN-γ by CD8^+^T cells upon PMA stimulation of BM-MNCs for 24 hrs when endogenously produced IL-10 was blocked by monoclonal antibody (iii & iv; *p=0.032, unpaired t test*).
