# Supplementary material for: Regulatory T Cells Suppress T Cell Activation at the Pathologic Site of Human Visceral Leishmaniasis
Source: PLoS One. 2012 Feb 8;7(2):e31551. doi: 10.1371/journal.pone.0031551 (PMC3275558; doi:10.1371/journal.pone.0031551)
Supplement: Figure S9 — Proliferation of non-Treg cells are inversely correlated with the frequency of FoxP3+ Treg cells in bone marrow (BM) of VL patients: Data shows analysis of proliferating Treg (Ki67+FoxP3+) and non-Treg (Ki67+FoxP3−) cells from (i & ii) blood and (iii & iv) BM of VL patients. Findings show decreased proliferation of CD4+FoxP3- cells at the disease sites (BM) compared to blood and sign of spontaneous proliferation of FoxP3+ Treg cells Gating is based on the isotype staining for Ki67 (i & iii). (DOC) [file pone.0031551.s009.doc]

**Figure S9**

**Figure S9: Proliferation of non-Treg cells are inversely correlated with the frequency of FoxP3^+^ Treg cells in bone marrow (BM) of VL patients:** Data shows analysis of proliferating Treg (Ki67^+^FoxP3^+^) and non-Treg (Ki67^+^FoxP3^-^) cells from **(i & ii)** blood and **(iii & iv)** BM of VL patients. Findings show decreased proliferation of CD4+FoxP3- cells at the disease sites (BM) compared to blood and sign of spontaneous proliferation of FoxP3^+^ Treg cells Gating is based on the isotype staining for Ki67 (i & iii).
